# Fatty acid unsaturation improves germination of upland cotton (*Gossypium hirsutum*) under cold stress

**DOI:** 10.3389/fpls.2024.1286908

**Published:** 2024-02-06

**Authors:** Lakhvir Kaur Dhaliwal, Junghyun Shim, Dick Auld, Rosalyn B. Angeles-Shim

**Affiliations:** Department of Plant and Soil Science, Davis College of Agricultural Sciences and Natural Resources, Texas Tech University, Lubbock, TX, United States

**Keywords:** imbibition, cell membrane, cumulative melting temperature, cold stress, seed germination, unsaturated fatty acids, recombinant inbred lines

## Abstract

**Introduction:**

The level of fatty acid unsaturation in seeds is one of the major determinants of cold germination ability, particularly in oilseeds. The presence of *cis* double bonds in unsaturated fatty acids creates bends that lowers their melting temperatures compared to saturated fatty acids. Unsaturated fatty acids with low melting points mobilize faster at low temperatures providing seeds with sufficient energy for germination.

**Methodology:**

To investigate the effects of fatty acid unsaturation on the ability of cotton seeds to germinate under cold conditions, four recombinant inbred lines (RILs) of cotton with unique fatty acid profiles were evaluated using a set of developmental and biochemical assays at 12°C (critically low temperature), 15°C (cardinal minimum temperature) and 30°C (optimum temperature). Furthermore, whole seed lipidome profiling using liquid chromatography with mass spectrometry was done to compare the lipid compositional changes at 12°C and 30°C after imbibing cotton seeds of all the six genotypes for 0 hours, 3 hours and 6 hours.

**Results and discussion:**

The RILs with higher unsaturation/saturation ratios registered robust germination performance, lower solute leakage, and optimum water uptake rates under cold stress. Imbibition at 30°C for 8 hours before cold exposure significantly improved the germination of cold sensitive genotypes, indicating that the first few hours of water uptake are critical for cold stress. Whole seed lipidome profiling of all the genotypes specifically associated cold germination ability with higher unsaturation levels of phospholipids during early imbibition. The presence of *cis* double bonds in phospholipids creates kinks that maintain the fluidity of cell membranes under low temperature. Membrane flexibility under cold conditions is essential for facilitating key germination events including membrane organization and respiration. The current results highlight the importance of fatty acid composition in cold germination ability of upland cotton.

## Introduction

The relative proportions of saturated to unsaturated fatty acids in seeds are significant determinants of germination ability under variable temperatures. In general, seeds of tropical plants that require warm temperature for germination tend to have lower unsaturated fatty acid contents compared to their temperate counterparts (Iba, 2002; Upchurch, 2008). Palm seed for example, which germinates best at 30-35°C, contains approximately 48% unsaturated fatty acids (Marcus, 2013; Costa et al., 2018). In contrast, sunflower (*Helianthus annus* L.) seeds, which have optimum germination at 12-15°C, possess approximately 89% unsaturated fatty acids (Akkaya, 2018). Examination of other species within the genus *Helianthus* growing under a climate gradient that extends from Texas to Canada demonstrated a strong correlation between cold germination ability and higher unsaturated fatty acid contents (Linder, 2000).

The reported effects of fatty acid composition on seed germination ability in relation to temperature are linked to the variable melting temperatures of saturated and unsaturated fatty acids (Shim et al., 2019). The sharp 30-degree bends present in each *cis* double bond of unsaturated fatty acids pushes the lipid molecules away from each other, considerably lowering the melting points of unsaturated fatty acids (Kanicky and Shah, 2002). For instance, the introduction of a single covalent bond in the saturated stearic acid (18:0) to turn it into a monounsaturated oleic acid (18:1) substantially reduces melting point from 69.3°C to 13.4°C (Linder, 2000). Under colder conditions, unsaturated fatty acids characterized by lower melting temperatures maintain a liquid crystalline phase, in contrast to saturated fatty acids that undergo a transition from a liquid crystalline to a more solid gel state. The quicker transition of saturated fatty acids into a solid state can seriously disrupt membrane fluidity, cellular homeostasis, molecular transport, cellular signaling and respiration under colder conditions. In *Escherichia coli* for example, cold-induced rigidity of mitochondrial membranes has been reported to hinder the movement of the electron transport carrier, ubiquinone. Consequently, electron transport decelerates, triggering the excessive generation of reactive oxygen species (ROS) that causes oxidative damage and death of cold-exposed cells (Gohrbandt et al., 2022).

Cotton is a tropical/subtropical plant species and therefore has inherent sensitivity to cold at all growth stages. Its seeds contain approximately 50% linoleic acid, 22–26% palmitic acid, 16–20% oleic and 2–3% stearic acid (Liu et al., 2009; Dowd et al., 2010; Dowd, 2015; Shim et al., 2019). Despite a relatively high level of overall unsaturation, cotton seeds have a cardinal minimum temperature of 15°C for germination. Temperatures below 15°C constitutes cold stress that negatively affects germination, starting from excessive cellular leakage (Sanchez et al., 2019; Dhaliwal et al., 2021). In a previous study, we identified recombinant inbred lines (RILs) of cotton that germinated robustly under temperatures below the cardinal minimum for the crop (15°C). Compared to conventional cultivars, the lines have higher linoleic acid (unsaturated fatty acid) but lower palmitic acid (saturated fatty acid) content (Shim et al., 2019). While the ability of the RILs to sustain germination under cold stress correlated with the potentially improved cellular energetics as conferred by the non-conventional fatty acid profile of the RILs, the hypothesis has yet to be confirmed by empirical data. Using four cotton RILs with unique profiles for the four major fatty acids present in cotton seeds, this research was undertaken to determine the effects of increased fatty acid unsaturation on the ability of seeds to germinate under cold stress. In addition, the effects of overall unsaturation/saturation content of seeds on the composition of membrane lipids following cold water imbibition were determined by lipid profiling.

## Materials and methods

### Plant materials

Seeds of two conventional cotton cultivars (SA 2580 and SC 9023) and four recombinant inbred lines (RILs) (FAM 1, FAM 2, FAM 3, FAM 4) with varying fatty acid profiles were used in the study (**Table 1**). The RILs were generated by intercrossing the M_5_ mutants AFIS 1-1422-A5 and SCM3-7-3-A3 and advancing the resulting F_1_ to F_8_ generation by single seed descent. The mutants were generated from the chemical mutagenesis of SA 2580 and SC 9023 and were initially selected for herbicide tolerance and fiber quality traits, respectively. At the M_5_ generation, the mutants were also determined to have low palmitic (18.10-18.70%) and high linoleic acid contents (56.80-58.70%) (Shim et al., 2019; Thompson et al., 2019). Sibs of the RILs have been reported previously to be robust germinators under cold stress (Shim et al., 2019).

**Table 1:** Fatty acid composition of cotton seeds used in the study.

Genotype	Fatty acids				CMT^a^ (°C)	US/S ratio^b^	Phenotype
	Palmitic acid (C16:0)	Stearic acid (C18:0)	Oleic acid (C18:1)	Linoleic acid (C18:2)			
	—————————% methyl esters————————						
FAM 1	18.50	2.00	18.80	61.50	12.74	3.92	LP/HL
FAM 2	18.40	2.80	21.90	57.70	13.88	3.76	LP/HL
FAM 3	16.20	4.50	39.50	39.70	17.18	3.83	LP/HO/LL
FAM 4	37.60	4.20	22.20	30.70	28.32	1.27	HP/HO/LL
SA 2580	23.50	2.40	17.10	53.70	16.29	2.73	normal
SC 9023	25.00	2.70	17.40	51.50	17.61	2.49	normal

a cumulative melting temperature.

b calculated by dividing unsaturated fatty acid content (oleic+linoleic) with saturated fatty acid content (palmitic+stearic).

LP, low palmitic acid; LL, low linoleic acid; HO, high oleic acid; HL, high linoleic acid; HS, high stearic acid.

### Fatty acid profiling of the RIL seeds

The fatty acid composition of the RIL seeds was analyzed from cotyledon tissues of each sample using gas chromatography (GC) after fatty acid methyl esterification (FAME) of extracted oils (Thompson et al., 2019). Briefly, seed tissues were sampled distally from the embryo and ground in 1 ml hexane. The extracted oil was transferred to a reacti-vial and separated from hexane by N_2_ evaporation of the solvent. The resulting clear, solid residue was dissolved in 1.5 ml FAME solution composed of 29.1 ml of 14% borontrifluoride in methanol (MeOH), 20 ml toluene and 50.9 ml MeOH. The mixture was heated at 90°C for 30 minutes, cooled, supplemented with 1.5 ml distilled water, and transferred to a larger test tube where it was rinsed twice with 1.5 ml hexane. Following N_2_ evaporations of the solvent, fatty acid methyl esters of the extracted oil were re-suspended in 1 ml hexane for GC analysis.

Samples at 2 µl were injected into an Agilent 6890 GC system with split inlet and FID detector operated at 300°C and fitted with a Supelco SP 2380 capillary column (30 m x 0.25 mm id- 0.20 um film thickness). Helium was used as the carrier gas at a linear velocity of 20 cm per second. The initial temperature was set to 200°C for 1 minute and then increased by 2°C per minute until it reached 214°C. After 3 minutes at 214°C, the temperature was further increased by 3°C per minute until it reached 230°C. The temperature was held at 230°C for 10 minutes to complete a run time of 26.3 minutes. Calibration of the GC system for the cotton FAMEs was performed using an RM-3 mixture as a standard.

Fatty acid detection was carried out using the Agilent Chem station for GC systems. Cumulative melting temperature (CMT) for each genotype was determined based on the four major fatty acids detected by GC in the seeds namely palmitic acid (C16:0), stearic acid (C18:0), oleic acid (C18:1) and linoleic acid (C18:2). CMT was calculated using the formula CMT = (% C16:0*62.78°C) + (% C18:0*69.44°C) + (% C18:1*13.33°C) + (% C18:2* -5°C)/100 (Thompson et al., 2019).

### Imbibition rate and water content assessment

To determine the effects of cold stress on the hydration rates of germinating cotton seeds, the water uptake patterns of the six genotypes were determined under normal (30°C) and cold temperature (12°C). Seeds were kept under ambient temperature and 60% relative humidity for 4 days to equilibrate moisture content to 10% (Usberti et al., 2006). Following equilibration, three seeds per genotype were weighed and allowed to re-hydrate for 24 hours at 30°C and 12°C under dark conditions. Imbibing seeds were weighed at 30-minute intervals. Changes in water uptake were calculated as percent change in the weight of imbibed seeds relative to the weight of dry seeds. The three phases of water uptake in cotton were established by plotting water uptake rates against the time elapsed.

### Electrolyte leakage measurements

Ten seeds per genotype were placed in a 50 ml falcon tube containing nanopure water for 1, 2, 3, 4 and 8 hours at a critically low temperature of 12°C, cardinal minimum temperature of 15°C and optimum temperature of 30°C. Initial electrical conductivity of the leachates was measured after each time point using a conductivity meter (Thermo Scientific, USA). Final electrical conductivity was obtained after boiling the seeds at 95°C for 45 minutes. Total conductivity expressed as relative electrolyte leakage (REL) was determined by dividing the initial with the final electrical conductivity. To avoid weight bias, REL values for each genotype were divided by the weight of the respective seeds used in the experiment.

### Lipid peroxidation analysis

The extent of oxidative damage on lipids following imbibition of seeds at 12°C, 15°C and 30°C for 3 and 6 hours was determined based on malondialdehyde (MDA) content expressed in nmol/ml (Heath and Packer, 1968; Hodges et al., 1999). Briefly, imbibed seeds were ground in liquid nitrogen and mixed in 1 ml of 0.1% trichloroacetic acid (TCA). The solution was then centrifuged at 13,000 rpm for 15 minutes and 400 µl of the supernatant was aliquoted into 0.5% thiobarbituric acid (TBA) solution prepared in 20% TCA. The resulting mixture was incubated in a water bath set at 85°C for 30 minutes and centrifuged at 13,000 rpm for 5 minutes. MDA content in the supernatant was estimated using a plate reader. Background noise in the absorbance readings was removed by path length correction where absorbance obtained at 600 nm wavelength was subtracted from absorbance obtained at 530 nm. The experiment was set up in duplicates to reduce the chances of experimental error.

### Cold germination screening

The germination performance of the experimental materials was evaluated at 12°C, 15°C and 30°C following a modified protocol (Shim et al., 2019; Liu et al., 2021). Briefly, twenty seeds per genotype were placed between three sheets of sterile filter paper contained in petri dishes and sprayed with approximately 6 ml of sterile, distilled water. The petri dishes were maintained for 14 days in a growth chamber programmed at different target temperatures.

The effects of hydropriming on the cold germination ability of the seeds were also examined under cold stress. Twenty seeds per genotype were plated as previously described and allowed to imbibe water for 8 h at 30°C before transferring them to 12°C or 15°C. After 14 days under cold stress, the plates were moved to normal temperature to determine the germination recovery of the seeds. All set-ups were prepared in duplicates.

Germination of seeds based on 2 mm radicle protrusion was monitored daily for 14 days and used to determine germination percentage (GP), mean germination time (MGT), mean daily germination (MDG), peak value (PV) and germination index (GI) for all genotypes. GP was derived by dividing the final number of germinated seeds by the total number of seeds and multiplying the quotient by 100. MGT was established following the formula MGT = Σ (n * m) ∕ Σn where n = number of seeds germinated on day m. MDG was established as the total germination percentage at the end of the test/total length of experiment period. PV was calculated by diving the cumulative percentage of full-seed germination on any day to the number of days to have that percentage (Djavansher and Pourbeik, 1976; Orchard, 1977). GI was derived using the formula GI = (14*n1) + (13*n2) + (12*n3) +………+ (1*n14), where n1, n2…. n14 are the seeds that germinated on the 1st through 14th day and 14, 13……and 1 are the weights given to the corresponding days of germination (Kader, 2005).

### Lipidome profiling

Based on the established water uptake patterns of germinating cotton seeds, temporal profiling of the whole seed lipidome of the six cotton genotypes was conducted using liquid chromatography with tandem mass spectrometry (LC-MS/MS). Five seeds per genotype that imbibed nanopure water at 12°C for 3 and 6 hours were ground into fine powder using liquid nitrogen. Ground tissues at 400 mg were mixed with 3 ml hot (70°C) isopropanol and 10 µl EquiSPLASH LIPIDOMIX Quantitative Mass Spec Internal Standard (Avanti Polar Lipids, USA). The resulting solutions were mixed thoroughly with 3 ml chloroform and 3 ml of 0.9% (w/v) NaCl and kept at 4°C overnight to allow clear phase separation. After 24 hours, 4 ml of the organic phase was transferred to a new tube, dried, and re-suspended in 50% methanol: 50% chloroform solution for lipid analyses.

Each sample at 5 µl volume was injected into a Q-Exactive HF (Thermo Fisher Scientific, USA) mass spectrometer ionized with a heated electrospray (HESI) probe. The HESI probe was set at a spray voltage of 3.8 kV in the positive (+) mode and 2.8 kV in the negative (−) mode, and capillary temperatures of 300°C (+) and 310°C (−), respectively. The lipid samples were chromatographically separated on a Vanquish LC system using an Acquity UPLC column (2.1 mm x 100 mm, BEH C8, 1.7 µm, 130 A) (Waters Corporation, MA, USA). Two solvents composed of water, 0.1% formic acid and 5 mM ammonium formate (Solvent A), and 85% methanol:15% isopropanol, 0.1% formic acid and 2 mM ammonium formate (Solvent B) were used to separate the lipids at a gradient of 28 minutes, flow rate of 400 µl/minute and column temperature of 50°C. Mass spectrometry analysis based on data-dependent acquisition mode with two scan events was carried out in both ion modes for every sample. The first scan event was a full MS scan of 250–1200 m/z at a mass resolution of 120,000 full width with an automatic gain control target of 1 × 10^6^ and a maximum injection time of 100 milliseconds. The first ten ions with the highest intensity identified during the first scan were scanned a second time at 30 x 10^3^ resolution.

Lipid molecules detected with LC-MS/MS were identified using the LipidSearch software v4.1. The extracted MS/MS spectra were examined *in silico* for exact matches in lipid spectral libraries. While the mass tolerance for the precursor and MS/MS products were maintained at 3 ppm and 5 mDa, respectively, MS/MS similarity score threshold was kept at five. The potential adductions involve -H, -Na and –NH4 for the positive ion mode (+) and hydrogen loss and formate for the negative ion (-) mode. Lastly, data in the ESI+ and ESI– modes were merged and the area under peaks for each lipid ID were used for the statistical analysis using Metaboanalyst 3.0.

Cold-induced alterations in the seed lipidome of all the genotypes were characterized based on changes in the content and composition of fatty acids that make up the lipid subclasses after 3 and 6 hours of imbibition at 12°C. Lipid unsaturation levels were calculated as unsaturated to saturated ratios (US/S) (Dogras et al., 1977). Graphical representation of the data was carried out using MS Excel and RStudio version 1.4.1106 (RStudio Inc, 2015).

### Statistical analysis

A *post hoc* Tukey’s test followed by a two-way analysis of variance (ANOVA) at p<0.05 significance level was carried out using R studio version 3.6.2 (RStudio Inc, 2015) to examine the differences among the genotypes for germination ability, electrolyte leakage, water uptake rates and lipid peroxidation under cold stress.

## Results

### Overall oil unsaturation and cumulative melting temperature depend on the relative proportions of fatty acids in seeds

The cotton RILs derived from the artificial hybridization of the M_5_ mutants AFIS 1-1422-A5 and SCM3-7-3-A3 produced genotypes with fatty acid profiles that deviate from those of the conventional cotton cultivars. FAM 1 and FAM 2 in particular, are characteristically high in linoleic acid but low in palmitic acid. FAM 3 has low linoleic and palmitic acids but high oleic acid content. FAM 4 contains high palmitic acid, high oleic acid but low linoleic acid content (**Table 1**). Based on the proportions of palmitic, stearic, oleic, and linoleic acids in the seeds, oil content in FAM 1, FAM 2 and FAM 3 were determined to have higher unsaturation levels compared to the conventional cultivars. Conversely, FAM 4 recorded an unsaturation level that is even lower than those of the conventional cultivars. The calculated cumulative melting temperature was lowest in FAM 1 (12.56°C) and FAM 2 (13.67°C), and highest in FAM 4 (27°C) (**Table 1**).

### Cotton genotypes with higher proportions of unsaturated fatty acids maintain optimum water uptake rate and low electrolyte leakage during cold water imbibition

**Figure 1:**
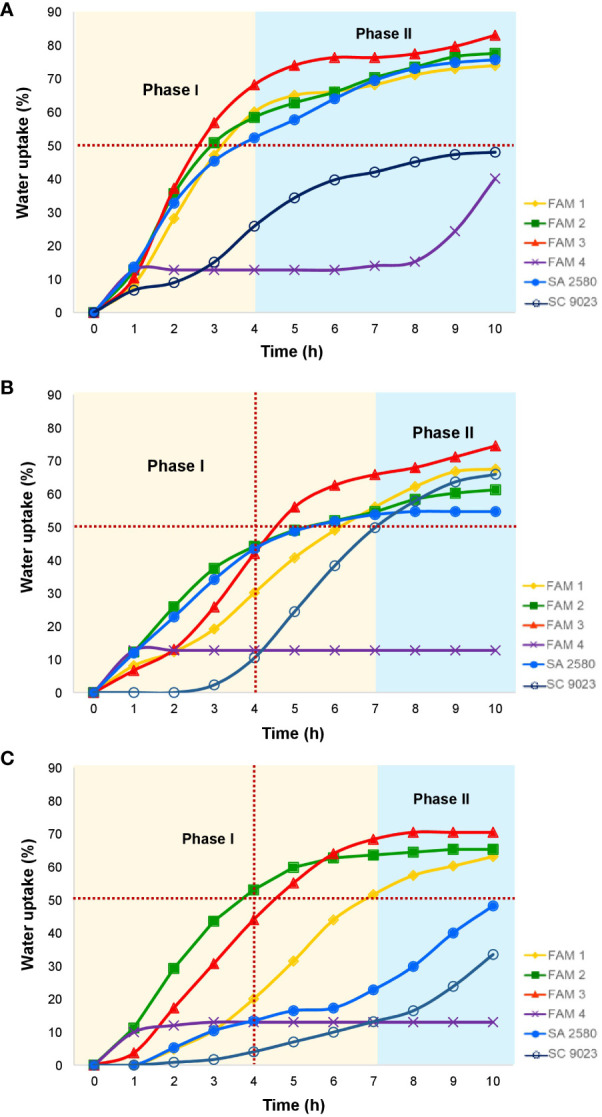
Water uptake patterns in cotton seeds imbibing water at **(A)** 30°C, **(B)** 15°C and **(C)** 12°C. Horizontal, red-dotted line indicates water uptake equivalent to 50% of dry seed weight.

Seeds of most plant species re-hydrate in a tri-phasic manner before they can germinate. Under normal temperature, seeds of all cotton genotypes except SC 9023 and FAM 4 exhibited a rapid initial water uptake that allowed seeds to reach ≥50-70% of their original dry weight within 3-4 hours of imbibition. In the next 6 hours, water uptake generally decelerated, although seeds continued to gain up to 70-80% of their original dry weight (**Figure 1A**). Lower temperatures significantly slowed down water uptake by the seeds, with all the genotypes except FAM 4 reaching ≥50% of their original dry weight after 7 hours of imbibition at 15°C (**Figure 1B**). Within the same duration at 12°C, only the cotton genotypes with higher fatty acid unsaturation namely FAM 1, FAM 2 and FAM 3 reached ≥50% of their original dry weight (**Figure 1C**).

**Figure 2:**
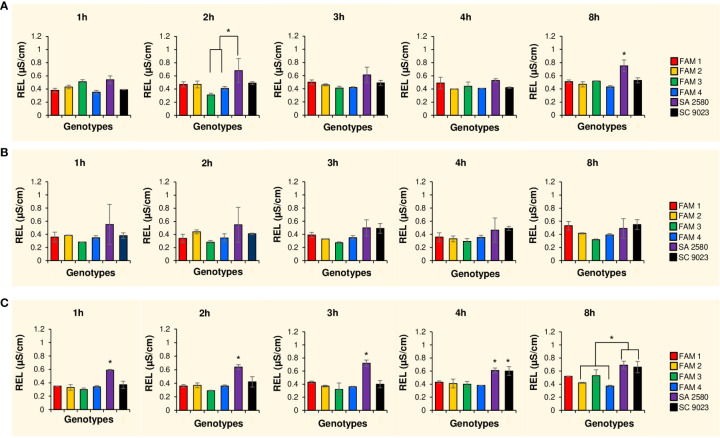
Histograms showing electrolyte leakage values after 1, 2, 3, 4 and 8 hours of imbibition at 30°C **(A)**, 15°C **(B)** and 12°C **(C)**. Error bars signify standard deviation between two replications. Asterisks indicate significantly different values at p<0.05.

To assess the effects of cold water imbibition on the integrity of the membranes, cellular leakage, measured in terms of relative electrical conductivity (REL), was assessed at various time points during imbibition. During the first 4 hours of rapid imbibition at normal temperature, seeds of the RILs and SC 9023 consistently showed lower cellular leakage compared to SA 2580. After 8 hours, cellular leakage in SA 2580 reached significantly higher levels compared to the rest of the genotypes (**Figure 2A**). At 15°C, the RILs also maintained generally lower values of REL compared to SA 2580 and SC 9023 throughout the 8-hour duration of water uptake (**Figure 2B**). At 12°C, the RILs were still able to maintain significantly lower cellular leakage compared to the conventional cultivars, especially during the first 4 hours of imbibition (**Figure 2C**).

**Figure 3:**
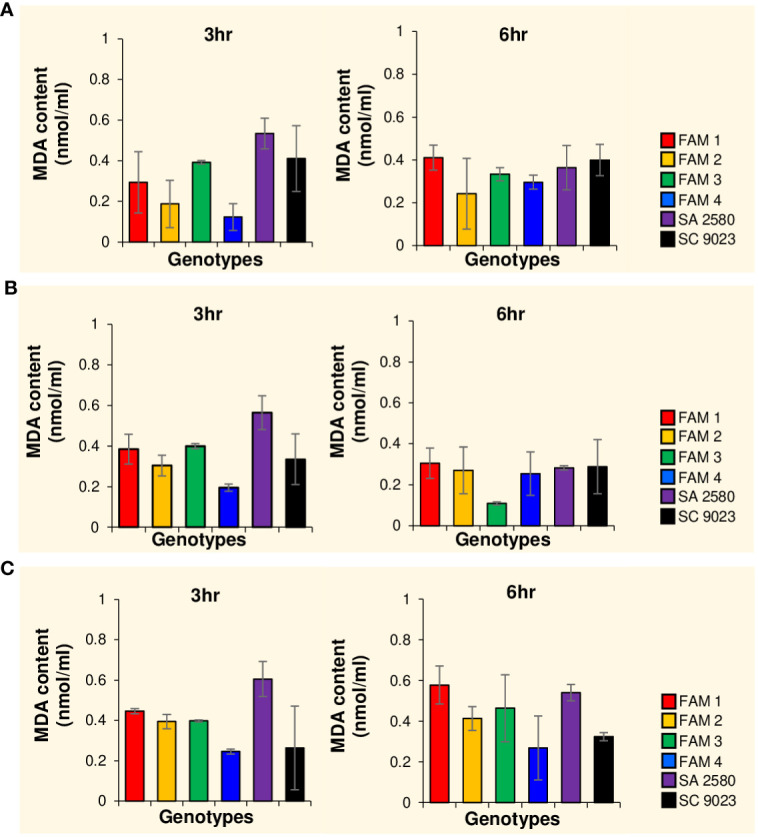
Histograms showing malondialdehyde (MDA) content in cotton seeds after 3 and 6 hours of imbibition at 30°C **(A)**, 15°C **(B)** and 12°C **(C)**. Error bars indicate standard deviation values for three replications.

To determine the degree of cold-induced, oxidative damage on the lipid components of the cell membrane, malondialdehyde (MDA) content was measured in cold-imbibed seeds. A general increase in MDA concentration was observed in FAM 1, FAM 2, FAM 4, and SA 2580 with decreasing temperature from 30°C to 12°C within the first 3 hours of imbibition. In contrast, FAM 3 maintained constant MDA content, whereas SC 9023 recorded decreasing values for the same parameter (**Figures 3A–C**). After 6 hours of water uptake, MDA content in FAM 1, FAM 2, FAM 3, and SA 2580 were higher in seeds that imbibed at 12°C than 30°C (**Figures 3A–C**).

### Cotton genotypes with higher proportions of unsaturated fatty acids have better cold germination ability

Seeds of all the tested genotypes showed high germination under normal temperature, ranging from 85% in SC 9023 to 100% in FAM 2. With decreasing temperature from 30°C to 12°C, germination percentage decreased, with SC 9023 seeds not germinating at all at 12°C. An overall general reduction in MDG, PV and GI, and an increase in MGT accompanied the decrease in germination in response to cold stress (**Table 2**).

**Table 2:** Mean values of germination parameters used to evaluate the germination ability of the RILs and conventional cultivars at three different temperatures viz., 12°C, 15°C and 30°C.

Germination parameter	Genotype	Temperature treatment				
		12°C	15°C	30°C	12°C^+^	15°C^+^
Germination percentage (GP; %)	FAM 1	81.97^a^	82.50^a^	92.11	95.00^a^	92.50^ab^
	FAM 2	60.00^a^	82.50^a^	100.00	97.50^a^	100.00^a^
	FAM 3	15.00^b^	75.00^a^	90.00	50.00^b^	95.00^ab^
	FAM 4	7.50^b^	84.17^a^	100.00	62.50^ab^	80.00^b^
	SA 2580	20.00^b^	65.00^a^	86.67	15.00^b^	100.00^a^
	SC 9023	0.00^b^	10.00^b^	85.00	32.50^b^	27.50^c^
Mean germination time (MGT)	FAM 1	8.53^a^	5.96	1.55^bc^	7.44^b^	3.70^cd^
	FAM 2	10.46^a^	5.19	1.10^c^	6.94^ab^	2.90^d^
	FAM 3	12.17^a^	4.10	1.50^bc^	8.07^ab^	5.69^ab^
	FAM 4	10.50^a^	6.05	1.43^bc^	11.12^a^	5.07^bc^
	SA 2580	11.88^a^	11.31	4.87^a^	10.67^a^	7.23^a^
	SC 9023	n/a	11.25	2.74^b^	10.89^a^	7.14^a^
Germination index (GI)	FAM 1	103.50^a^	147.50^a^	235.50	144.00^a^	203.50^a^
	FAM 2	57.50^ab^	161.00^a^	278.00	157.50^a^	242.00^a^
	FAM 3	8.50^b^	163.50^a^	243.00	69.00^b^	177.00^b^
	FAM 4	5.50^b^	143.00^a^	273.00	50.50^b^	159.00^b^
	SA 2580	12.50^b^	48.00^b^	263.50	13.00^b^	155.50^b^
	SC 9023	0.00^b^	13.00^b^	209.50	27.50^b^	43.50^c^
Mean daily germination (MDG)	FAM 1	5.86^a^	5.89^a^	6.58	4.75^a^	6.61^ab^
	FAM 2	4.29^a^	5.89^a^	7.14	4.88^a^	7.14^a^
	FAM 3	1.07^b^	5.36^a^	6.43	2.50^bc^	6.79^ab^
	FAM 4	0.54^b^	6.01^a^	7.14	3.13^ab^	5.72^b^
	SA 2580	1.43^b^	4.64^a^	6.19	0.75^c^	7.14^a^
	SC 9023	0.00^b^	0.72^b^	6.07	1.63^bc^	1.96^c^
Peak value (PV)	FAM 1	7.15^a^	5.89^bc^	27.13	9.31^a^	7.75^b^
	FAM 2	4.29^b^	8.57^ab^	60.00	8.92^a^	20.00^a^
	FAM 3	1.11^c^	13.75^a^	37.50	3.85^b^	8.64^b^
	FAM 4	0.70^c^	7.46^ab^	41.67	4.68^ab^	6.67^b^
	SA 2580	1.48^bc^	4.64^bc^	11.61	1.11^b^	7.42^b^
	SC 9023	0.00^c^	0.83^c^	7.20	2.43^b^	2.41^c^

^+^With hydropriming treatment.

Values within a column followed by different letters indicate significant differences at p<0.05.

Across genotypes, FAM 1 and FAM 2 maintained >50% germination even under 12°C, which translated into significantly higher average values for GP, GI, MDG and PV, and lower average values for MGT compared to the rest of the experimental materials. At the cardinal minimum temperature of 15°C, all RILs have either significantly or numerically higher average values for GP, GI, MDG and PV, and lower mean values for MGT. No considerable differences were observed among genotypes for the mean values of all the five parameters at 30°C (**Table 2**).

Hydropriming for 8 hours at 30°C prior to cold exposure of seeds facilitated a more uniform and faster radicle emergence in both the RILs and obsolete cultivars at 12°C and 15°C, with higher GP, GI, MDG and PV, and lower MGT.

### Cold imbibition altered the membrane composition of cotton seeds

**Figure 4:**
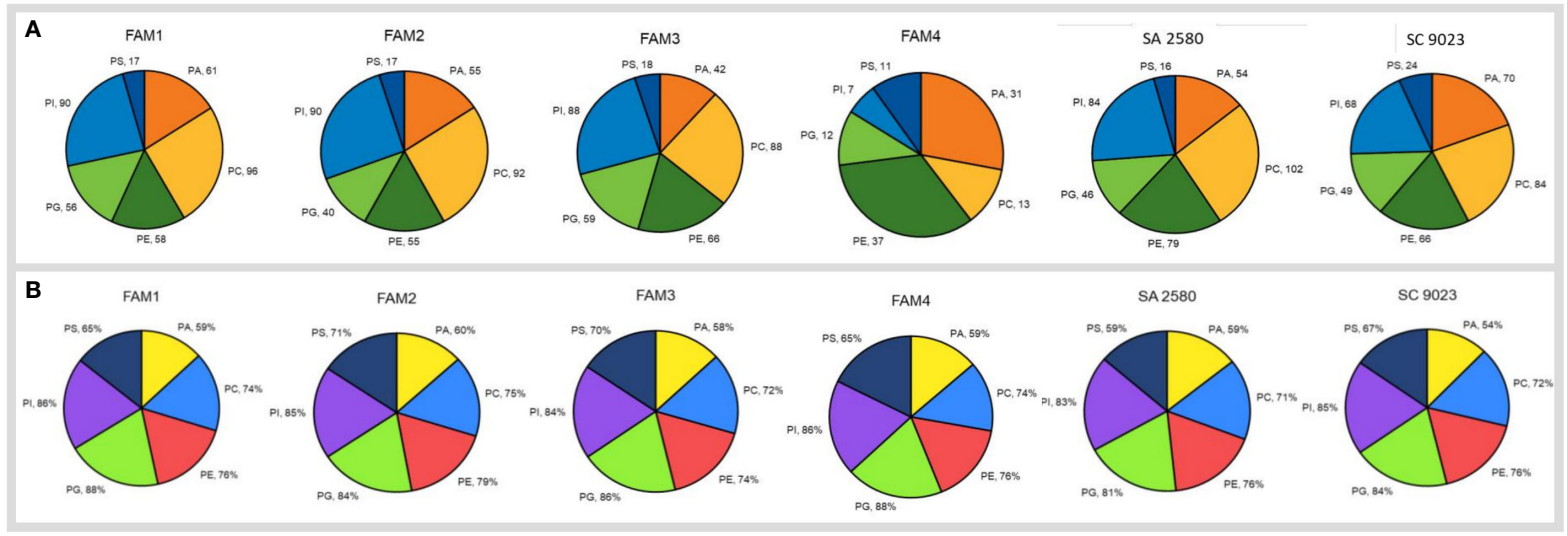
Pie charts in **(A)** represent the number of different glycerophospholipid sub-classes detected in the dry seeds of six genotypes. Pie-charts in **(B)** show combined percentage of five major fatty acids including palmitic acid (16:0), stearic acid (18:0), oleic acid (18:1), linoleic acid (18:1) and linolenic acid (18:2) in the dry cotton seeds of six different genotypes. The glycerophospholipids are abbreviated as: PA, phosphatidic acid; PC, phosphatidylcholine; PE, phosphatidylethanolamine; PG, phosphatidylglycerol; PI, phosphatidylinositol; PS, phosphatidylserine.

A total of 378 lipid molecules in dry seeds of FAM 1, 349 in FAM 2, 361 in FAM 3, 111 in FAM 4, 381 in SA 2580 and 361 in SC 9023 belonging to six subclasses of membrane lipids or glycerophospholipids were identified (**Figure 4**). The glycerophospholipid subclasses detected in the seeds were phosphatidic acid (PA), phosphatidylcholine (PC), phosphatidylethanolamine (PE), phosphatidylglycerol (PG), phosphatidylinositol (PI) and phosphatidylserine (PS). Among the lipid subclasses, PC made up the largest fraction in the dry seeds of all genotypes except FAM 4, which was composed largely of PE. Although the fatty acid composition of the molecular species comprising the different glycerophospholipids was highly variable (**Supplementary Table S1**), five fatty acids viz., palmitic acid (16:0), stearic acid (18:0), oleic acid (18:1), linoleic acid (18:2) and linolenic acid (18:3) were the most abundant. The combined proportion of these five major fatty acids belonging to different glycerophospholipids is provided in **Figure 4B**. Each of the glycerophospholipids contained at least 58% fraction of these five major fatty acids. The individual content of the five major fatty acids in the dry seeds of all the genotypes are presented in **Supplementary Table S2**.

**Figure 5:**
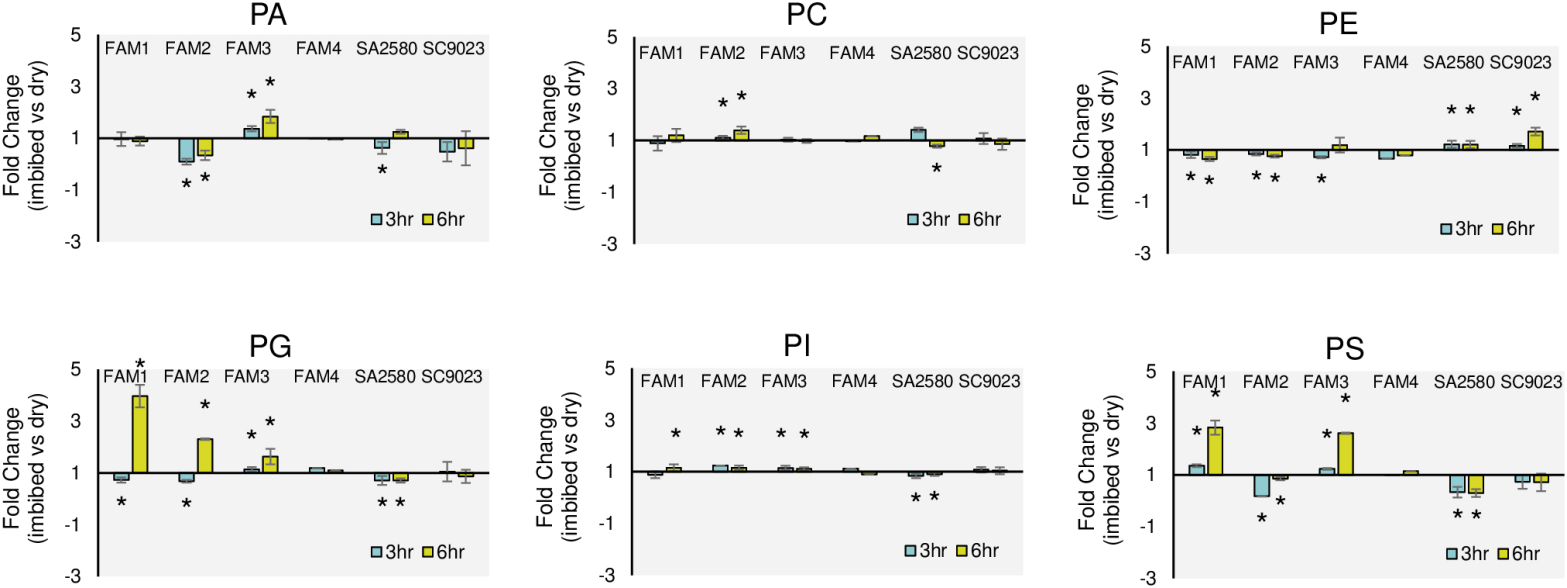
Fold changes in the unsaturation/saturation ratios (US/S) of glycerophospholipids after 3 and 6 hours of imbibition at 30°C and 12°C relative to dry seeds. Asterisks indicate significant fold change in US/S at p< 0.05. Error bars indicate standard deviation values for three replications.

To unravel the cold-induced changes in membrane lipid unsaturation of germinating cotton seeds, the unsaturation/saturation ratios (US/S) in dry and imbibed seeds of all the genotypes were calculated based on the proportion of the five major fatty acids. Analysis of unsaturation content showed that imbibition for 3 hours at cold temperature increased the unsaturation of PS in FAM 1; PC and PI in FAM 2; PA, PC, PI, PG and PS in FAM 3; PG, PI and PS in FAM 4; PC and PE in SA 2580; and PC, PE, PG and PI in SC 9023 (**Figure 5**). Extended cold imbibition up to 6 hours increased the unsaturation content of PC, PG, PI, and PS in FAM 1; PC, PG and PI in FAM 2; PA, PE, PI, PG and PS in FAM 3; PC, PG and PS in FAM 4; PA and PE in SA 2580; and PE and PI in SC 9023 (**Figure 5**).

## Discussion

Germinating seeds uptake water in a tri-phasic manner (Turner et al., 2021). The first phase is characterized by a rapid influx of water (i.e. imbibition) that is facilitated by the low water potential of mature, dry seeds. The second phase is defined by a hydration plateau (phase II) as the seed transitions from a quiescent to a metabolically active state. The third phase (phase III) is marked by a secondary increase in water uptake following the protrusion of the radicle through the seed coat and the elongation of the embryonic axis. In the present study, phase I of water uptake in germinating cotton seeds lasts up to 4 hours under normal temperature. Within this timeframe, the seeds absorbed water up to ≥50% of their dry weight. At 12°C, seeds of the conventional cultivars, as well as FAM 4 imbibed water at slower rates compared to FAM 1, FAM 2, and FAM 3 (**Figure 1**). Based solely on the fatty acid profiles of the experimental materials, the higher proportions of unsaturated fatty acids in FAM 1, FAM 2, and FAM 3 could have facilitated optimum water uptake rates even under cold stress. Interestingly, FAM 4 and SC 9023 imbibed slowly even under the normal temperature of 30°C. Neither genotype did not even reach the 50% water uptake margin throughout the 10-hour duration of the experiment. SC 9023 was bred specifically for the cold-prone areas of Texas High Plains where chilling fronts commonly occur during the planting season for upland cotton (Hinze et al., 2015). It is possible that seeds of this cultivar acquired traits like hard-seededness to slow down its water uptake to avoid imbibitional chilling injury. Testa of some seeds contains specific structures that control the imbibition process (Bewley et al., 2013).

Cold imbibition exacerbates solute leakage during germination. Cellular leakage during cold stress mainly involves the loss of electrolytes such as potassium ions. A simple way to quantify the amount of electrolytes leaked from the seeds is to measure the change in electrolytic conductivity of water used in seed imbibition. Changes in electrical conductivity are indicative of the relative quantity of cells that were unable to avoid solute leakage during cold water imbibition (Demidchik et al., 2014). Three genotypes named FAM 1, FAM 2, and FAM 3 with higher unsaturation content in their seeds recorded lower REL relative to SC 9023 (**Figure 2**). Interestingly, FAM 4 and SC 9023 also registered lower REL values compared to the other conventional cultivars. This may be attributed to the slow water uptake of SC 9023 and FAM 4 up to 7 and 10 hours after imbibition at 12°C, respectively. Although the genotypes with higher unsaturation content recorded significantly lower REL values compared to SA 2580 for the first few hours of imbibition under cold stress, the difference among them after 8 hours of imbibition was not as pronounced, indicating that the first few hours of imbibition are critical under cold stress. The results of the water imbibition experiment (**Figure 1**) confirm the well-established fact that phase 1 of water uptake, which is also known as the imbibition phase, is a cold-sensitive step of the germination process (Bewley et al., 2013; Yu et al., 2015; Noblet et al., 2018).

Overproduction of reactive oxygen species (ROS) in response to several abiotic stresses such as cold, drought and salinity is known to cause lipid peroxidation (Shen et al., 2000; Mittler et al., 2004; Gong et al., 2005; Celikkol et al., 2010; Gong et al., 2011). Lipid peroxidation causes structural and functional alterations in cells which disrupt membrane permeability, as well as the proper functioning of several membrane proteins (Simon, 1974; Bailly et al., 1996). To measure the effects of fatty acid composition on the extent of lipid peroxidation in seeds under cold stress, malondialdehyde (MDA) content was measured after imbibing seeds of all genotypes for 3 and 6 hours at 12°C, 15°C and 30°C. Oxidative damage in response to cold imbibition was evidenced by the higher MDA content detected in seeds of almost all the genotypes during phase I of water uptake at 12°C compared to 30°C. This confirms the well-established effects of low temperature stress on membrane stability (Suzuki and Mittler, 2006; You and Chan, 2015; Hussain et al., 2018; Xia et al., 2019). However, no correlation was established between MDA content and fatty acid composition of the cotton seeds imbibed for 3 and 6 hours. The lack of correlation might be attributed to the increased sensitivity of unsaturated fatty acids towards lipid peroxidation. Although genotypes with higher unsaturation content exhibit cold tolerance, their susceptibility to lipid peroxidation confounds the MDA results, particularly when comparing genotypes with varying fatty acid levels. These results are similar to the findings of Noblet et al. (2018), which showed non-significant differences in the MDA content of cold-sensitive and cold-tolerant corn seeds after 24 hours of imbibition at 10°C.

The better water uptake rates and low solute leakage in FAM 1 and FAM 2 seeds with higher unsaturation content translated to a better, faster, and more uniform germination under cold stress (**Tables 1**, **2**). In contrast, the reduced water uptake rates and higher solute leakage in FAM 4 (HP/HO/LL) and conventional cultivars having lower unsaturated fatty acid proportions led to abnormal metabolic processes in seeds, germination delay, and poor uniformity. Interestingly, FAM 3 recorded poor germination at 12°C despite the higher unsaturation levels in its seeds (**Table 1**). Relative to FAM 1 and FAM 2, FAM 3 possesses lower linoleic acid:oleic acid ratio (**Table 1**). At low temperatures, germination rate depends on the faster metabolization of polyunsaturated fatty acids such as linoleic and linolenic than the monounsaturated oleic acid (Jungman and Schubert, 2000). In peanut, germination percentage at lower temperatures (16°C and 14°C) decreased with increased oleic/linoleic acid ratio (Jungman and Schubert, 2000). Other studies have also reported poor seed vigor, poor germination, and reduced yield of peanut genotypes with high oleic acid content relative to genotypes with normal levels of oleic acid (Sun et al., 2014; Upadhyaya et al., 2014; Bera et al., 2019).

Hydropriming for 8 hours at 30°C prior to cold exposure of seeds facilitated a more uniform and faster radicle emergence in all the genotypes at 12°C and 15°C. The conventional cultivars registered as high as 1400% improvement in germination performance, indicating that the first few hours of warm imbibition are critical for cold stress. These results support previous findings on the positive effects of hydropriming on the ability of cold-sensitive genotypes to germinate under low temperature stress (Płażek et al., 2018; Zhu et al., 2021).

Early imbibition involves an extensive biosynthesis of phospholipids necessary for membrane repair and replacement (Pollock and Toole, 1966). During phase I, for instance, phospholipids quickly reorganize themselves from a leaky hexagonal II configuration to a semi-permeable lamellar configuration in the cell membrane (Christiansen and Thomas, 1969; Buitink and Leprince, 2008). While the hexagonal II configuration is an ill-configured and leaky structure, lamellar configuration is a non-leaky, semi-permeable structure. If water imbibition begins while the seed is in hexagonal II configuration, solutes may leak out from the cell, leading to seed metabolic dysfunction, embryo death and poor germination (Dhaliwal and Angeles-Shim, 2022). To restore cellular homeostasis, phospholipids quickly reorganize membranes into a lamellar configuration upon rehydration. The successful completion of membrane reorganization has been reported as the most crucial event, serving as a pre-condition for the majority of the other cellular events during germination (Yu et al., 2015). At low temperatures, the lipid components of cell membranes are forced into a highly rigid organization that results in the loss of membrane fluidity (Simon, 1974). Consequently, this delays the re-organization of cell membranes into a bi-layer, exacerbating solute leakage and ultimately leading to radicle tip abortion and embryo death (Sanchez et al., 2019).

Cold acclimating plants, however, tend to increase lipid unsaturation, which is needed for membrane fluidity, re-organization and signaling (Kates et al., 1984; Stubbs and Smith, 1984). Phospholipidome studies on maize showed that cold exposure for 24 hours at 10°C increased the unsaturation content of the phospholipids PC, PE, PG and PI in seeds, which facilitated their germination even at low temperatures (Noblet et al., 2018). In the present study, the better water uptake rates as well as low solute leakage observed in FAM 1, FAM 2 and FAM 3 during early imbibition indicate towards the integrity of membranes even under colder conditions.

To specifically investigate whether cold tolerant genotypes exhibit elevated membrane lipid unsaturation during early imbibition, lipidome profiling was done after imbibing cotton seeds for three and six hours at low and normal temperatures. Interestingly, no correlation was found between the cold tolerance levels and the unsaturation/saturation ratios of membrane lipids in the dry seeds of all the six genotypes (**Supplementary Table S3**). To explore it furthermore, cold induced changes in the unsaturation content were investigated by calculating fold changes in the unsaturation/saturation ratio during early imbibition compared to dry seeds. FAM 1, FAM 2, and FAM 3 recorded a significant increase in the unsaturation of the major phospholipid classes PC, PG, PI, and PS upon imbibition. A significantly high, 4-fold change in the unsaturation of fatty acids comprising PG was recorded in the seeds of FAM 1, FAM 2, and FAM 3 following 6 hours of imbibition under cold stress (**Figure 5**). In contrast, the conventional cultivars significantly increased the unsaturation content of only PE after 3 and 6 hours of imbibition (**Figure 5**). The unsaturation content of the other membrane lipids either decreased significantly or increased in negligible amounts. In the conventional cultivars and FAM 4, which showed higher solute leakage and poor germination performance at 12°C, no significant increase in unsaturation content was recorded in any of the membrane lipids even after 6 hours of continuous cold exposure.

The elevated unsaturation levels observed in the imbibed seed of FAM 1, FAM 2 and FAM 3 could be attributed to the hydrolysis of unsaturated TAGs during early imbibition. Enzymatic lipolysis hydrolyzes TAGs to release free fatty acids which then enter peroxisomes for β-oxidation. However, the activation of free fatty acids into acyl-CoAs in the cytoplasm is a prerequisite before their entry into peroxisomes, influencing the content and composition of acyl-CoA pools and consequently impacting the composition of membrane lipids (Yu et al., 2021). It might be possible that the unsaturated oil of FAM 1, FAM 2 and FAM 3 enriched acyl CoA pool with unsaturated fatty acids resulting in their accumulation in membrane lipids during early imbibition.

In the present study, the enhanced unsaturation content of the phospholipids in FAM 1, FAM 2 and FAM 3 conferred fluidity to the membranes leading to better uptake rates and reduced solute leakage even at the critically low temperature of 12°C. In particular, increased unsaturation content of PG ensured crucial chloroplast functions necessary for the successful emergence of radicle (Xu et al., 2020). In a previous study, chilling tolerance of sensitive tobacco (*Nicotiana tabacum*) plants was improved by increasing the desaturation of PG through the overexpression of the *glycerol-3-phosphate acyltransferase* gene (Murata et al., 1992). In contrast, failure to enhance desaturation content in seeds of the sensitive conventional cultivars and FAM 4 in response to cold disrupted cell membrane organization, leading to excessive solute leakage. This ultimately led to poor germination performance.

## Data availability statement

The original contributions presented in the study are included in the article/**Supplementary Material**. Further inquiries can be directed to the corresponding author.

## Author contributions

LK: Data curation, Investigation, Writing – original draft. JS: Data curation, Investigation, Writing – review & editing. DA: Investigation, Resources, Writing – review & editing. RA-S: Conceptualization, Funding acquisition, Supervision, Writing – original draft, Writing – review & editing.
